# Efficacy and Safety of Wheat Grass in Thalassemic Children on Regular Blood Transfusion

**DOI:** 10.7759/cureus.2306

**Published:** 2018-03-11

**Authors:** Amit S Mutha, Kalpana U Shah, Aarti A Kinikar, Balasaheb B Ghongane

**Affiliations:** 1 Department of Pharmacology, Grant Medical College & Sir J.J.Group of Hospitals, Mumbai; 2 Department of Pharmacology, B. J. Government Medical College & Sassoon General Hospitals, Pune; 3 Department of Pediatrics, B. J. Government Medical College & Sassoon General Hospitals, Pune

**Keywords:** blood transfusion, wheat grass, thalassemia

## Abstract

Background: In thalassemia, mutations either in alpha or beta chain synthesis results in low hemoglobin (Hb). Wheatgrass has been used for many years for health purposes. Some reports suggest the beneficial effect of wheatgrass on transfusion requirements. Folic acid is also known to play an important role in several biochemical reactions. In some patients with thalassemia, the supplementation of folic acid is useful.

Objective: To evaluate the efficacy and safety of wheatgrass in children with thalassemia receiving chronic blood transfusions.

Material and methods: In this randomized prospective study, 69 children with thalassemia were divided into the wheatgrass group and the control group (no wheatgrass). Both groups received a regular blood transfusion and folic acid. The treatment duration was 18 months. Anthropometric parameters, number of transfusions, and amount of blood transfused were compared within and between the groups at the end of the therapy. Clinical examinations, laboratory investigations, and ultrasounds for liver and spleen span were performed at the baseline and then every six months till 18 months. Adverse effects (if any) were noted on every visit. Quality of life (QOL) was evaluated before and at the end of the study using a questionnaire.

Results: Sixty-nine (study group (n=45; mean age 6.35 ± 2.65 yrs); control group (n=24; 4.86 ± 2.77 yrs)) patients were enrolled, of which 12 from the study group and three from the control group did not complete the study. The difference in liver size within the wheatgrass group was significant (P <0.021) only at 18 months but not in the control group at any time point. The difference in spleen size was significant within the wheatgrass group (P<0.005) as well as the control group (P<0.001) at 18 months only. The difference in serum ferritin levels was not significant between the two groups. The increase in serum ferritin levels at the end of the study was significant in the control group when compared to the baseline (P<0.01). There was no difference in the average number of transfusions or in the blood transfusion requirement between the two groups. The difference in the QOL at the start and end was significant in the wheatgrass group (P<0.05).

Conclusion: Wheatgrass appears to play a promising role in children with thalassemia receiving chronic blood transfusions.

## Introduction

Hemoglobinopathies are one of the major health problems in India [1]. Beta-thalassemias are common in the Indian population with the average rate of affected people ranging between 3%-4%. The rates differ between different communities and regions in India [1-3].

The data from western India reported a prevalence of up to 9.5% [3]. Children with beta-thalassemia major have a poor quality of life [4-5]. Similarly, thalassemia is also associated with cardiac complications [6]. Bone marrow transplantation is the treatment of choice for thalassemia major; however, due to limitations especially related to donors, it is not commonly performed in many patients. Patients need lifelong blood transfusions in the absence of bone marrow transplantation [5,7]. Folic acid plays an important role in several biochemical reactions and the maturation of red blood cells [8]. Many pharmacological options have also been tried but without consistent response [7]. Therefore, clinicians are always in search of safer and effective alternative options for the treatment of thalassemia.

A recent study showed a significant reduction in serum ferritin level with non-pharmacological therapy, including nutrition, exercise, and a praying program in patients with beta-thalassemia major [9]. A herbal approach is another area under investigation. Wheatgrass has been studied in the treatment of pancytopenia [10], hematological toxicity related to chemotherapy in cancer patients [11], and in thalassemia [12-13]. In Indian studies, wheatgrass has been shown to reduce the requirements of blood transfusion [12-13]. The effect of wheatgrass on other laboratory, clinical, and ultrasound parameters in patients with thalassemia are largely unknown.

Objective

The objective of this study was to evaluate the effects of wheatgrass in thalassemia patients on regular blood transfusion and folic acid therapy with respect to a rise in hemoglobin and maintenance, fetal hemoglobin (HbF), serum ferritin, frequency and requirement of blood transfusion, spleen and liver span, and hematological parameters.

## Materials and methods

In this randomized, prospective, open-label study conducted in the thalassemia day care center, department of pediatrics, at a tertiary care hospital in Western Maharashtra, parents of 100 children were approached for participation. Patients of β-thalassemia major and intermedia requiring regular blood transfusion therapy were included. Patients who had undergone splenectomy during the study period, patients receiving iron chelation or any known fetal hemoglobin (HbF)-inducing drug, active liver disease, suffering from malaria, serum creatinine > 1.7 mg%, any chronic disease, such as tuberculosis, human immunodeficiency virus (HIV) or hepatitis B surface antigen (HbsAg) reactive, or those with any adverse drug reaction were excluded from the study. Patients who satisfied enrollment criteria were randomized into the wheatgrass and control groups. Randomization was carried out by using the table of random numbers. Patients in the wheatgrass group were given eight tablets per day for the first fortnight, followed by 12 tablets per day divided into three equal doses for 18 months. Patients in the control group were not given wheatgrass tablets. A folic acid tablet (5 mg/day) was taken regularly by patients in both the groups, as folic acid is needed in the maturation of red blood corpuscles (RBCs), and in the Indian population, folic deficiency is rampant due to a predominantly vegetarian diet.

A general and systemic examination was performed during each visit of the wheatgrass group. A chest X-ray, electrocardiogram, and serum ferritin were done at the baseline and at the end of the study. A complete blood count (CBC), peripheral blood smear, HbF, erythrocyte sedimentation rate (ESR), liver function test (LFT), serum creatinine, and ultrasound were done at the baseline and at six monthly intervals. Anthropometric parameters, i.e. weight, height, and head circumference; pre- and post-transfusion hemoglobin, amount of blood transfused, interval between two subsequent transfusions, and the side effects of blood transfusion were recorded at each visit. Blood transfusion requirement (ml/kg/year) was assessed at the end of the study period and compared with the requirements of the previous one year. Adverse effects, if any, were noted on every visit. Quality of life before and at the end of the study was evaluated by a specifically designed questionnaire [14-15]. The study was initiated after approval from the Institutional Ethics Committee and consent from the parent/lawful guardian.

Statistical analysis

Continous data are presented as the mean and standard deviation whereas categorical data are presented as number and percentages. The chi-square test was applied to assess the gender-wise distribution of patients in the two groups. Serum ferritin, HbF, CBC, LFT, serum creatinine, and spleen and liver span were analyzed by using the students’ ‘t’ test between the groups. The average number of transfusions, blood transfusion requirement, and pre-transfusion hemoglobin of all patients in the wheatgrass and control groups one year before the start of the study were calculated from their previous records at the center and were compared with similar observations during the study period. Data were analyzed using the students’ ‘t’ test. For within the group comparison of various parameters, analysis of variance (ANOVA) for repeated measures was applied.

## Results

**Figure 1:**
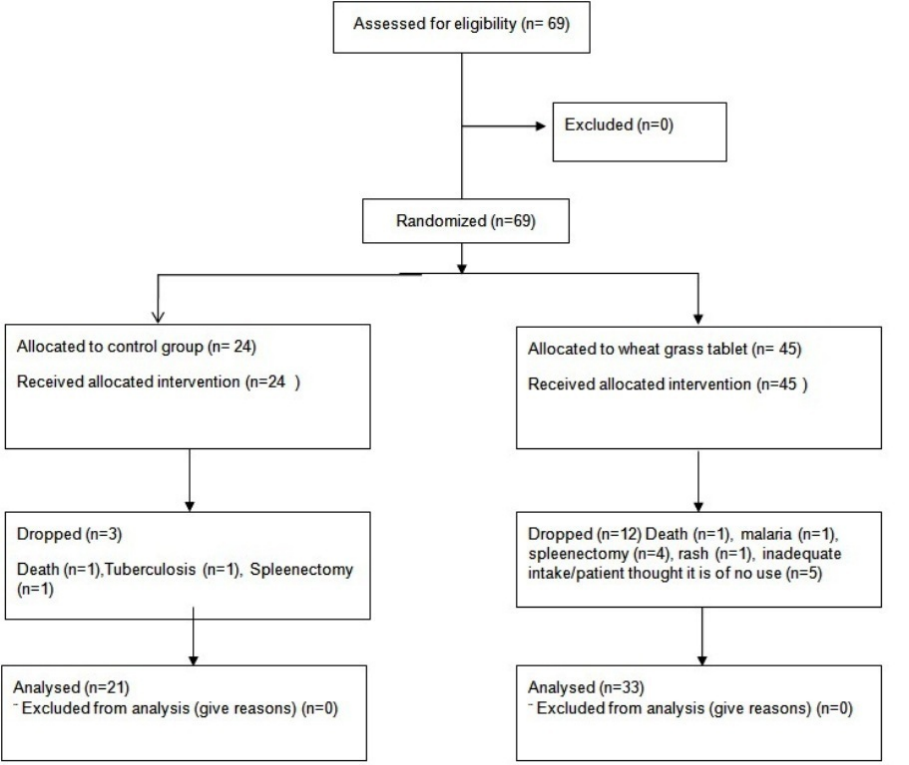
Study flow diagram of the whole sample

A total of 69 (wheatgrass tables (n=45) or control group (n=24)) patients were enrolled. Twelve (26.67%) and three (12.5%) patients from the wheatgrass and control group, respectively, did not complete the study. The reasons for not completing the study are listed in Figure 1.

The difference in gender distribution was not significant in the wheatgrass group but was significant (P<0.05) in the control group. The difference in age was significant between the two groups (P<0.05; Table 1).

**Table 1:** Demographic characteristics

Characteristics	Wheatgrass (n=33)	Control (n=21)
Male/ Female	16 / 17	14 / 7* *p<0.05
Age (years)	6.35 ± 2.65	4.86 ± 2.77* *p<0.05

The difference in weight and height was significant (P<0.05), but the difference in head circumference was not significant between the two groups. There was a significant difference (P<0.05) in the height and weight before and after the study period within the two groups (Table 2).

**Table 2:** Effect on anthropometric characteristics

	Wheat Grass (n=33)		Control (n=21)	
	Before	After	Before	After
Weight (kg)	15.18 ± 4.29*	17.38 ± 4.3 *	12.42±3.84	14.84±4.07*
Height (cm)	102.88 ± 13.27*	109.95 ± 11.99*	92.86± 15.98	100.9.± 14.68*
Head Circumference (cm)	49.53 ± 1.56	49.11 ± 8.16	48.62.± 2.06	47.64.± 10.09
*P<0.05				

On clinical examination, the difference in the liver size between the two groups was not significant. However, the difference in the liver size within the wheatgrass group was significant (P<0.021) only at 18 months but not in the control group at any time point. Similarly, the difference in the spleen size between the two groups was not significant. However, the difference in the spleen size was significant within the wheatgrass group (P<0.005) as well as the control group (P<0.001) at 18 months only. The difference in HbF levels was not significant between the two groups. However, the increase in HbF levels was significant within the wheatgrass group (P<0.03) at 18 months compared to the baseline whereas in the control group, it was not significant. The difference in hematocrit, RBC count, MCV, MCH, and serum creatinine was not significant between the two groups as well as within the two groups. The difference in ESR was significant at the baseline (P<0.05) and at 12 months (P<0.05) between the two groups but not significant within the two groups. The difference in MCHC was significant between the two groups at the end of the study (P<0.03) but not significant within the two groups (Table 3).

**Table 3:** Effect on clinical, laboratory, and ultrasound findings in different time periods

	Wheatgrass (n=33)				Control (n=21)			
	Baseline	6 months	12 months	18 months	Baseline	6 months	12 months	18 months
Liver span (cm)	4.27± 1.66	5.3 ± 2.1	4.88 ± 2.15	4.97 ± 2.2 (P<0.021)	3.33 ± 1.83	4.36 ± 2.39	4.24 ±1.14	4.12 ± 1.05
Spleen span (cm)	7.3± 3.79	7.62 ± 4.08	8.38 ± 4.72	8.91 ± 4.31(P< 0.005)	5.67 ± 3.1	6.31 ± 3.26	8.43 ± 4.03	7.79 ± 4.05 (P<0.001)
Hemoglobin F (%)	9.11 ±8.27	8.36 ±8.71	9.73 ±7.70	10.91 ±9.64(P<0.03)	11.46 ±11.16	11.67 ±13.15	10.05 ±8.55	9.71 ±8.67
Haematocrit (%)	17.35 ±5.10	17.52 ±5.43	17.93 ±3.90	18.01 ±4.69	18.55 ±6.02	18.72 ±5.87	18.93 ±4.30	18.64 ±5.09
Red Blood Cell count (× 106 / µL) levels	2.52 ±1.80	2.21 ±0.63	2.28 ±0.42	2.32 ±0.85	2.31 ±0.73	2.11 ±0.67	2.28 ±0.52	2.24 ±0.58
Erythrocyte Sedimentation Rate (mm at 1hour)	17.00 ±2.59 (P<0.05)	16.36 ±3.50	16.97 ±2.20 (P<0.05)	16.52 ±2.85	15.14 ± 2.78	15.00 ± 3.11	15.33 ± 2.31	15.71 ± 1.76
Mean Corpuscular Volume (fl)	80.27 ±11.00	81.40 ±9.02	80.60 ±8.41	79.63 ±9.86	78.67 ±7.93	84.09 ±7.68	82.68 ±6.78	82.01 ±6.99
Mean Corpuscular Hemoglobin (pg)	26.84 ±2.90	27.41 ±2.88	27.30 ±2.33	26.99 ±2.47	26.35 ±3.07	27.35 ±3.15	27.16 ±2.66	26.77 ±2.96
Mean Corpuscular Hemoglobin Concentration (g/dl)	33.94 ±2.01	33.89 ±2.36	33.76 ±1.62	33.72 ±1.80 (P<0.05)	33.24 ±1.80	32.78 ±2.23	32.87 ±2.09	32.66 ±1.58
SGPT (IU/L)	49.64 ±36.52	49.70 ±18.25	45.42 ±17.41 (P<0.05)	43.42 ±19.42	36.24 ±17.08	43.57 ±11.24	36.90 ±11.59	36.81 ±18.19
SGOT (IU/L)	65.58 ±30.66	59.03 ±31.15 (P<0.001)	52.15 ±24.77	53.76 ±27.75 (P<0.001)	52.95 ±27.67	35.90 ±17.30	41.48 ±15.07	42.48 ±16.92 (P<0.001)
Serum Bilirubin (mg /dl)	0.91 ±0.52	0.87 ±0.33 (P<0.05)	0.85 ±0.23 (P<0.05)	0.83 ±0.23	0.76 ±0.22	0.71 ±0.16	0.73 ±0.12	0.73 ±0.16
Serum Creatinine (mg /dl)	0.72 ±0.24	0.69 ±0.15	0.67 ±0.12	0.70 ±0.15	0.67 ±0.21	0.65 ±0.15	0.67 ±0.13	0.68 ±0.14
Spleen span (cm) on Ultrasonography	13.61 ±3.68	14.13 ±3.42 (P<0.05)	14.34 ±3.95	14.56 ±4.11	11.85 ±2.46	12.24 ±2.65	12.82 ±2.50	12.66 ±2.39
Liver span (cm) on Ultrasonography	12.78 ±2.16	12.77 ±2.35	12.72 ±2.28	12.93 ±2.30	12.49 ±1.75	12.89 ±1.17	13.20 ±1.87	13.77 ±1.17

The difference in SGPT was not significant between the two groups except at 12 months (P<0.05). The difference in SGPT within two groups was not significant. Similarly, the difference in SGOT was not significant between the two groups except at six months (P<0.001). The difference in SGOT was significant within the wheatgrass (P<0.005) and control (P<0.001) groups.

The difference in serum bilirubin was not significant between the two groups at the baseline and 18 months but was significant at six (P<0.05) and 12 (P<0.04) months. The difference was not significant between the two groups.

The difference in spleen span on ultrasound was not significant at the baseline between the two groups. However, a significant difference was seen at six months (P<0.05), but this difference was not seen at 12 and 18 months between the two groups. The difference in liver span was not significant between the two groups.

**Figure 2:**
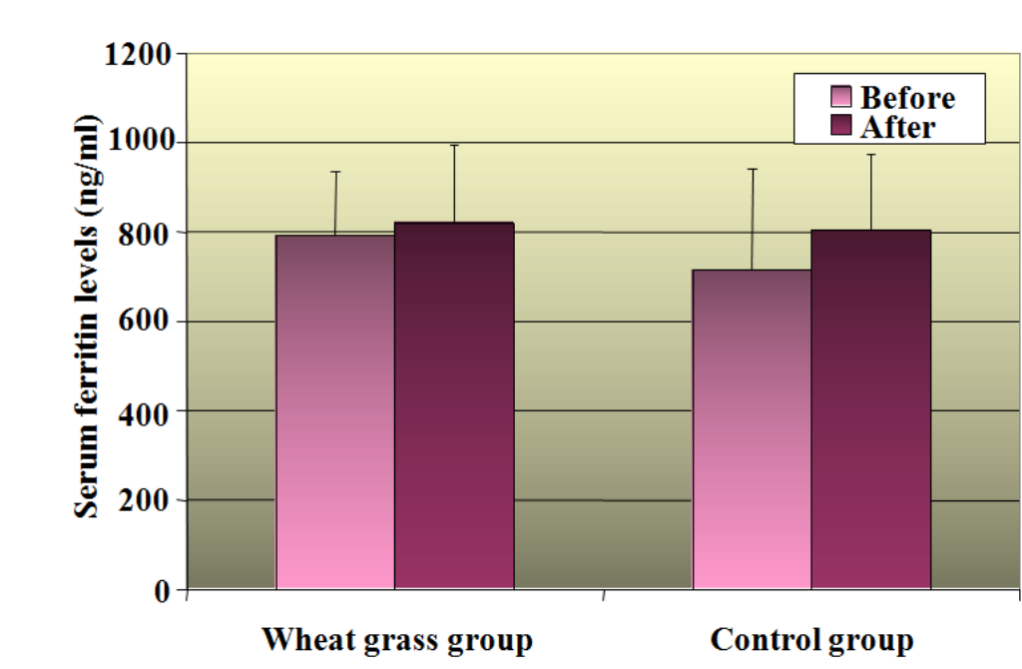
Comparison of serum ferritin between two groups

The difference in serum ferritin levels was not significant between the two groups. Similarly, it was also not significant when ‘before’ and ‘after’ the study period levels were compared in the wheatgrass group whereas the difference was significant (P<0.01) in the control group. Increase in serum ferritin levels at the end of the study was significant in the control group when compared with the baseline. (P<0.01; Figure 2).

**Figure 3:**
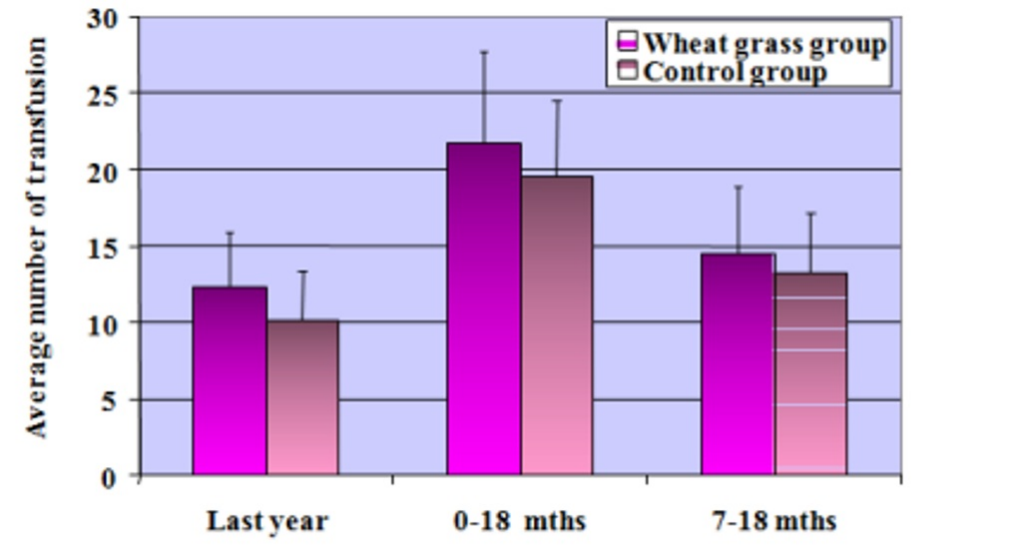
Comparison of transfusion between two groups (average number)

In both the groups, the difference in the average number of transfusions was significant when last years’ average number of transfusions was compared (P<0.05), but the difference was not significant between the two groups (Figure 3).

**Figure 4:**
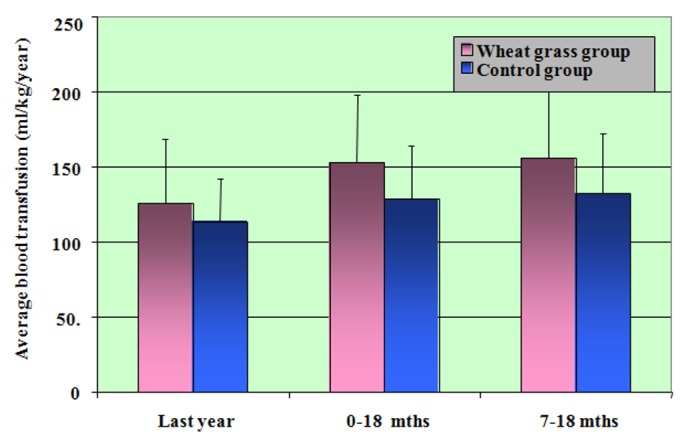
Comparison of transfusion between two groups (ml/kg/year)

The difference in blood transfusion requirements (ml/kg/year) was not significant during the last year between the two groups (Figure 4).

The difference in the quality of life [15] at the start of the study was not significant but at the end of the study, it was significant between the two groups (P<0.05). The difference in the quality of life at the start and end was significant within the wheatgrass group but not in the control group (P<0.05; Table 4).

**Table 4:** Effect on quality of life

	Wheatgrass (n=33)		Control (n=21)	
	Before	After	Before	After
Quality of life questionnaire score	55.21 ± 10.24	58.03 ± 8.28 (P<0.05)	52.52 ± 7.15	52.38 ± 7.05

## Discussion

Wheatgrass extract is considered an adjuvant in thalassemia although research in this area is in its infancy. The postulated mechanism of wheatgrass extract is the rapid absorption of chlorophyll and action at the cellular level in the bone marrow, assisting in heme production [16]. Recently, in the murine model, the iron chelator property of wheatgrass extract and its purified compound, mugineic acid, has been proved [17]. The use of the wheatgrass in the treatment of the iron overloaded disorder could also be justified because of the antioxidative property of methyl pheophorbide, a compound isolated from wheatgrass [18].

There are not many studies of wheatgrass in thalassemia. Marwaha et al. [13] demonstrated the effect of wheatgrass in chronically transfused children. Wheatgrass juice directly prepared from wheatgrass sprouts harvested on the seventh day was used in the published study. We used wheatgrass in the form of readymade dehydrated powder converted into tablets, with an objective of improving patient compliance. The treatment was given for 18 months. Twelve out of 45 patients in the wheatgrass groups did not complete the study due to various reasons, the major reasons being splenectomy, an inability to take wheatgrass tablets due to palatability, and no subjective benefit perceived by parents. The completion rate in the wheatgrass group in our study was 66.67%, better than the reported rate of 42% [13]. One patient each died in the study group and the control group. The number of patients undergoing splenectomy in the study group was higher than in the control group. Due to the smaller sample size, we did not examine the statistical difference between the two groups.

The significant difference in the mean age observed in the two groups in our study may be contributed to the unequal number of patients in both the groups. There was no significant difference in gender-wise distribution between the two groups. We observed a significant difference in height and weight both before and after the study period between the two groups possibly due to the unequal number of patients in the two groups and a significant difference in the mean age of the two groups. A significant difference in height and weight within the two groups, before and after the study period, could be because of the normal growth of the child.

Liver and spleen sizes are indicators of extramedullary hemopoeisis and thus indirectly indicate the adequacy of blood transfusion. Liver size may also indicate iron overload. There was no significant change in the size of the liver and spleen between the two groups. However, there was a significant difference in liver size within the wheatgrass group and a highly significant difference in spleen size within the wheatgrass and control groups. There was a significant increase in serum ferritin levels in the control group at the end of the study. The serum ferritin level was not increased in the wheatgrass group, indicating its possible role in iron overload states. The findings suggest that wheatgrass could at least be of use as an adjuvant treatment with chelating agents and may help to reduce their dose and, possibly, their side effects. However, further studies are required to ascertain the exact role. In the present study, there was no significant change in blood transfusion requirement; hence, the role of wheatgrass in the intestinal absorption of iron needs to be investigated. Similarly, other factors, such as the effect of diet, should be assessed in detail. In our study, there was no significant difference in HbF levels between the two groups in the beginning. However, HbF levels significantly increased within the wheatgrass group at the end of the study but not so in the control group. In two children with sickle cell anemia and β-thalassemia taking wheat grass tablets over five weeks, a substantial increase in HbF and a decrease in HbS has been reported. In the study by Marwaha et al. [13], they considered the first six months as the washout/neutral period, considering that some time is required for action. We considered total 18 months since the time patients started taking tablets. There was a significant difference in the average number of transfusions during the last year between the two groups, possibly due to the difference in the mean age of the two groups. No significant difference was seen between the two groups when the number of transfusions for the 0-18 months' and 7-18 months' time period was compared. We also found an increase in blood transfusion requirements in almost all patients in both groups, statistically significant when the 0-18 months' time period was compared, but it was not significant in the 7-18 months' time period. An increase in blood transfusion may be explained by the fact that the weight and activity of the growing child increased the transfusion requirement, at least in younger children. Two studies reported a reduction in blood transfusion requirements [12-13]. One study reported a reduction in blood transfusion requirement by >25% in eight patients (50%), with a decrease of >40% in three of these cases [13]. Another study from All India Institute of Medical Sciences showed that wheatgrass therapy given for one year does not reduce the transfusion requirement in patients with thalassemia [12]. We also could not demonstrate a reduction in blood transfusion requirement. In our study, wheatgrass tablets were well tolerated without significant adverse effects or clinically relevant laboratory changes. One patient in the study group had rashes over his body; wheatgrass was stopped immediately. However, the definite causality of rashes with wheatgrass is not proven.

At the start of therapy, a few patients may have adverse gastrointestinal events, which improve after some days. Considering this, patients were advised to take eight tablets for a fortnight followed by 12 tablets in divided doses. For better palatability, the tablets were given with milk, honey, or fruit juice (other than citrus fruits) or with water initially, for a few days. There was no significant difference in hematological indices and serum creatinine values. However, liver enzymes in the wheatgrass group were increased as compared to the control group. The exact clinical significance of this is not known. A long-term study or further evaluation to determine the cause of these changes is required. There was a significant difference in the quality of life in patients on wheatgrass as compared to the controls, suggestive of some subjective benefit.

Unequal demographic distribution, a small number of patients and a short follow-up period are some of the limitations of our study. Dose escalation studies need to be performed to find out the effective dose of wheatgrass tablets. An estimation of the active principle of the wheatgrass is another potential area for active research in the future.

## Conclusions

Treatment with wheatgrass tablets maintains serum ferritin levels and increases HbF levels in thalassemic children receiving chronic blood transfusions. However, there is no reduction in the frequency and blood transfusion requirement. Wheatgrass tablets also improve the QOL of children with thalassemia. A longer study with a larger number of patients would be required to confirm the usefulness of wheatgrass therapy in patients with transfusion-dependent anemia.
